# Resection of Liver Metastasis from Lacrimal Gland Adenoid Cystic Carcinoma: A Case Report and Review of the Literature

**DOI:** 10.70352/scrj.cr.26-0340

**Published:** 2026-09-29

**Authors:** Takashi Sugio, Satoshi Takada, Shotaro Tokuraku, Junichi Ogawa, Naoto Nishida, Hiroaki Horio, Ryohei Takei, Kaichiro Kato, Mitsuyoshi Okazaki, Yasuhito Iwao, Shinichi Nakanuma, Isamu Makino, Masayuki Takahira, Hiroko Ikeda, Shintaro Yagi

**Affiliations:** 1 Department of Hepato-Biliary-Pancreatic Surgery and Transplantation, Kanazawa University, Kanazawa, Ishikawa, Japan; 2 Department of Ophthalmology, Kanazawa University, Kanazawa, Ishikawa, Japan; 3 Department of Diagnostic Pathology, Kanazawa University, Kanazawa, Ishikawa, Japan

**Keywords:** lacrimal gland, adenoid cystic carcinoma, hepatectomy

## Abstract

**INTRODUCTION:**

Adenoid cystic carcinoma (ACC) is a rare malignancy that most commonly occurs in the major salivary and lacrimal glands. Although distant metastases frequently occur in the lungs, bones, and brain, liver metastases are uncommon, and the optimal management of metastatic lesions remains unclear. Here, we report a case of liver metastasis from a lacrimal gland adenoid cystic carcinoma (LGACC) treated with surgical resection.

**CASE PRESENTATION:**

A 38-year-old man underwent tumor resection for LGACC 6 years previously. Bilateral pulmonary metastases were detected 2 years after the primary surgery and treated with partial lung resection. Two liver metastases were identified during follow-up imaging. Because curative resection was considered feasible, the patient underwent laparoscopic right anterior sectionectomy of the liver. The postoperative course was uneventful, and the patient remained recurrence-free for 11 months after hepatectomy.

**CONCLUSIONS:**

Surgical resection may be a potential treatment option for selected patients with liver metastases from ACCs.

## Abbreviations

ACC
: adenoid cystic carcinoma
DWI
: diffusion-weighted imaging
LGACC
: lacrimal gland adenoid cystic carcinoma

## INTRODUCTION

ACC is a relatively rare malignant tumor that most commonly arises in the salivary or lacrimal glands, although it can also originate in other organs such as the esophagus and bronchi.^1)^ ACC is characterized by a strong tendency for perineural invasion, indolent growth, and a high propensity for hematogenous distant metastasis.^1)^ The lungs, bones, and brain are the most common sites of metastases, whereas liver metastases are rare. Consequently, the natural history of liver metastasis, the role of therapeutic interventions, including surgical resection, and their impact on prognosis remain poorly understood. Here, we report a case of liver metastasis from a LGACC treated with surgical resection, with a favorable short-term outcome.

## CASE PRESENTATION

A 38-year-old man underwent tumor resection for left-sided LGACC 6 years previously. The pathological diagnosis was pT2N0M0 pStage II according to the Union for International Cancer Control 8th edition, with evidence of perineural invasion (**Fig. 1**). To reduce the risk of local recurrence, proton beam therapy (75 GyE in 35 fractions) was administered 2 months after surgery, and the patient subsequently underwent routine follow-up imaging. Two years after the initial surgery, bilateral pulmonary metastases were detected and treated with a partial lung resection (**Fig. 2**). Six months before admission, fluorodeoxyglucose-PET (FDG-PET) revealed abnormal FDG uptake in the liver, and MRI revealed 2 nodular lesions.

**Fig. 1:**
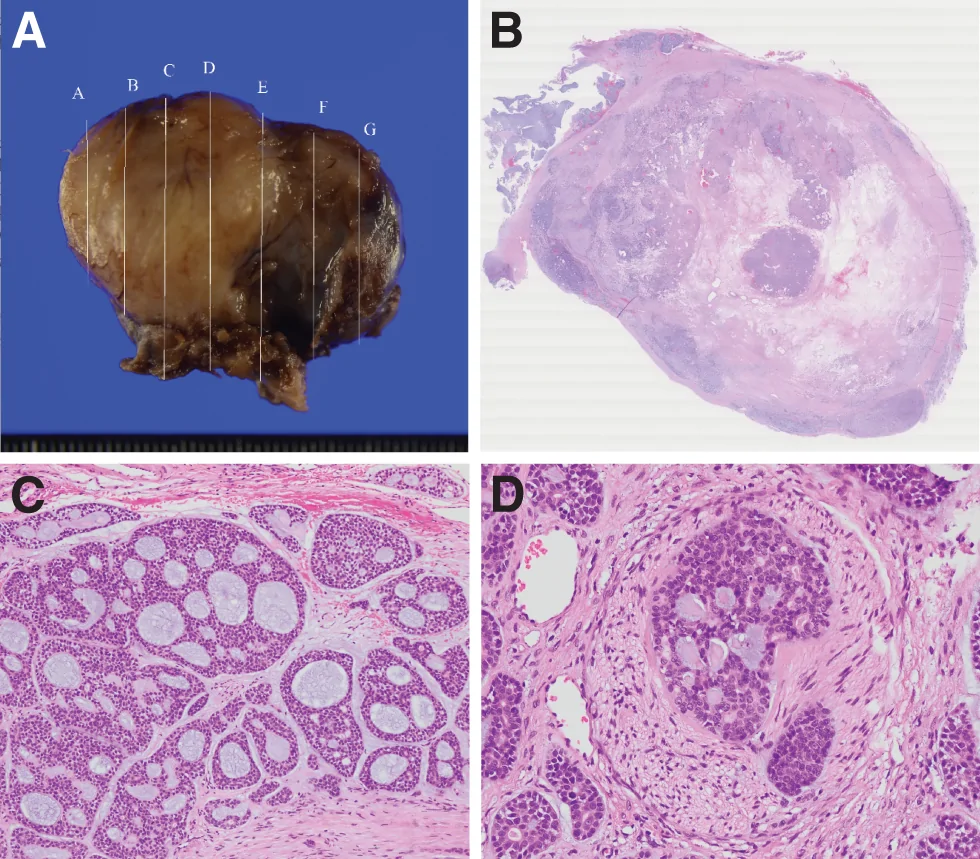
Resected specimen and pathological findings of lacrimal gland adenoid cystic carcinoma. (**A**) Gross findings (The white lines labeled A–G indicate the sectioning planes), (**B**) H&E, ×40, and (**C**) H&E, ×100: The tumor measured 3.7 cm in greatest dimension and was composed of relatively small, uniform cells arranged in cribriform, trabecular, and tubular architectures surrounded by abundant hyalinized stroma. (**D**) H&E, ×200: Perineural invasion was observed. H&E, hematoxylin and eosin

**Fig. 2:**
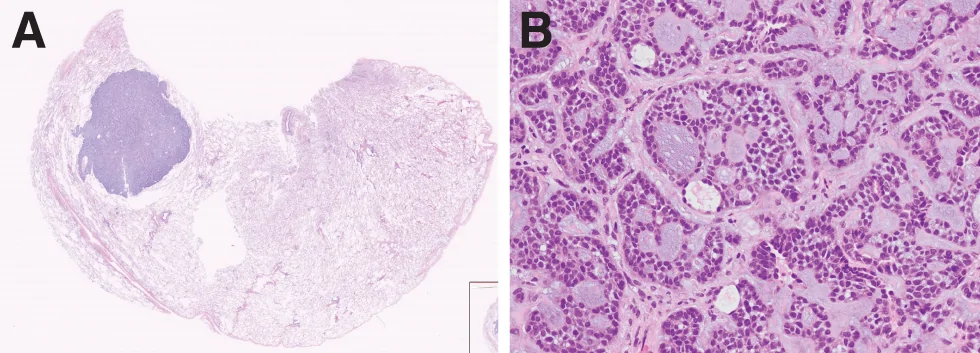
Pathological findings of the pulmonary metastases. (**A**) H&E, ×100. (**B**) H&E, ×200. Similar to the primary tumor, the metastatic lesion was composed of tumor cells arranged in cribriform, trabecular, and tubular patterns. H&E, hematoxylin and eosin

Physical findings: Height 168.7 cm, weight 58.3 kg, and BMI 20.5 kg/m^2^.

Tumor markers: Carcinoembryonic antigen 2.2 ng/mL, carbohydrate antigen 19-9 6.0 U/mL, and alpha-fetoprotein 2.0 U/mL (all within the normal range).

Contrast-enhanced abdominal MRI (**Fig. 3**): A 20-mm lesion was identified at the border between segments 7 and 8 (S7/8), and a 9-mm lesion was identified at the border between segments 5 and 8 (S5/8). Both lesions exhibited early contrast enhancement, high signal intensity on DWI, and decreased gadolinium ethoxybenzyl diethylene-triamine pentaacetic acid uptake.

**Fig. 3:**
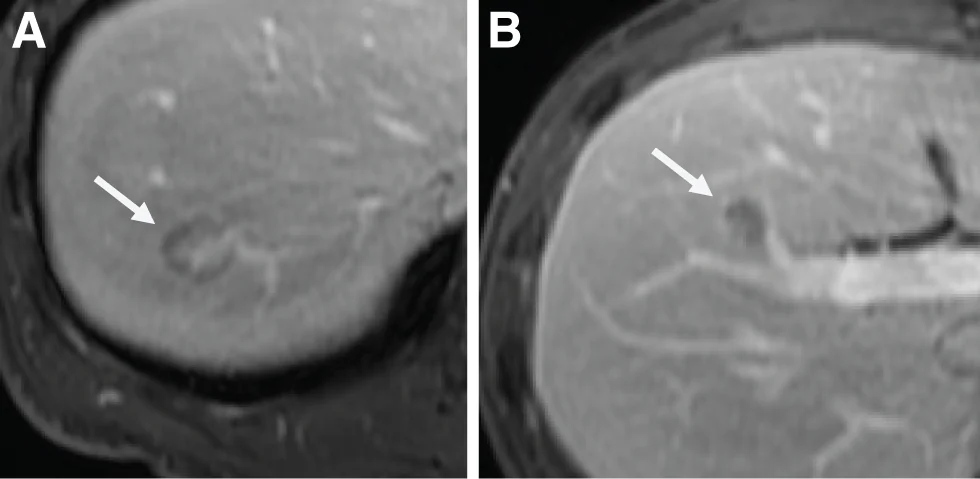
Contrast-enhanced abdominal MRI of the liver. (**A**) An enhanced 20-mm mass at the border between S7/8 (arrow). (**B**) An enhanced 9-mm mass at the border between S5/8 (arrow). S5/8, segments 5 and 8; S7/8, segments 7 and 8

FDG-PET (**Fig. 4**): FDG uptake (maximum standardized uptake value [SUVmax] 2.9) was noted in the S7/8 lesion. No other abnormal FDG uptake suggestive of metastatic disease was observed. There were no findings suggestive of another primary malignancy or underlying liver disease predisposing to primary hepatic malignancy.

**Fig. 4:**
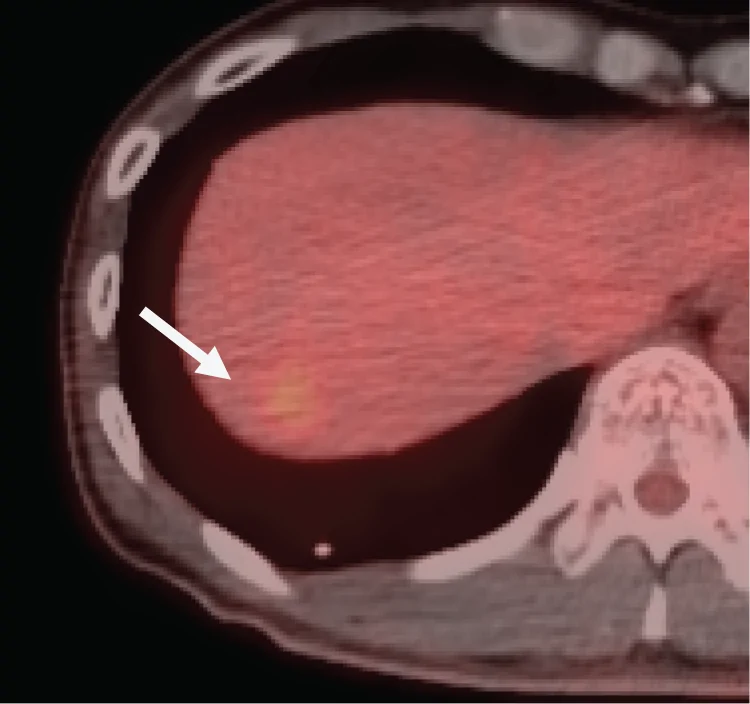
FDG-PET. The image showed FDG accumulation (SUVmax: 2.9) at the border between S7/8. No abnormal FDG uptake was observed elsewhere in the body. FDG-PET, fluorodeoxyglucose-PET; S7/8, segments 7 and 8; SUVmax, maximum standardized uptake value

Based on the overall clinical findings, the liver lesions were clinically diagnosed as metastases from LGACC. Since both lesions were considered resectable and no other active metastatic lesions were identified, surgical resection was planned. Because the S5/8 lesion was located near the origin of the anterior sectional branch, a right anterior sectionectomy was required to achieve complete tumor resection. The resection was extended to include a portion of S7, allowing simultaneous resection of the S7/8 lesion.

Intraoperative findings: The tumors were not visible on the liver surface but were visualized using US. The procedure was performed as planned and completed without any complications. The operative time was 476 min, and blood loss was 300 mL.

Histopathological findings: Macroscopically, 2 well-defined grayish-white nodules measuring 20 and 9 mm were identified (**Fig. 5**). Microscopically, the tumors consisted of cells with hyperchromatic nuclei and a high nuclear-to-cytoplasmic ratio arranged in cord-like or reticular patterns. The cells contained tubular or sieve-like structures filled with pale basophilic substances (**Fig. 6**). Immunohistochemical examination demonstrated positivity for c-kit (CD117), MYB, and p63, supporting the diagnosis of ACC. The Ki-67 labeling index was high, indicating high proliferative activity (**Fig. 7**). These findings were consistent with those of metastatic LGACC.

**Fig. 5:**
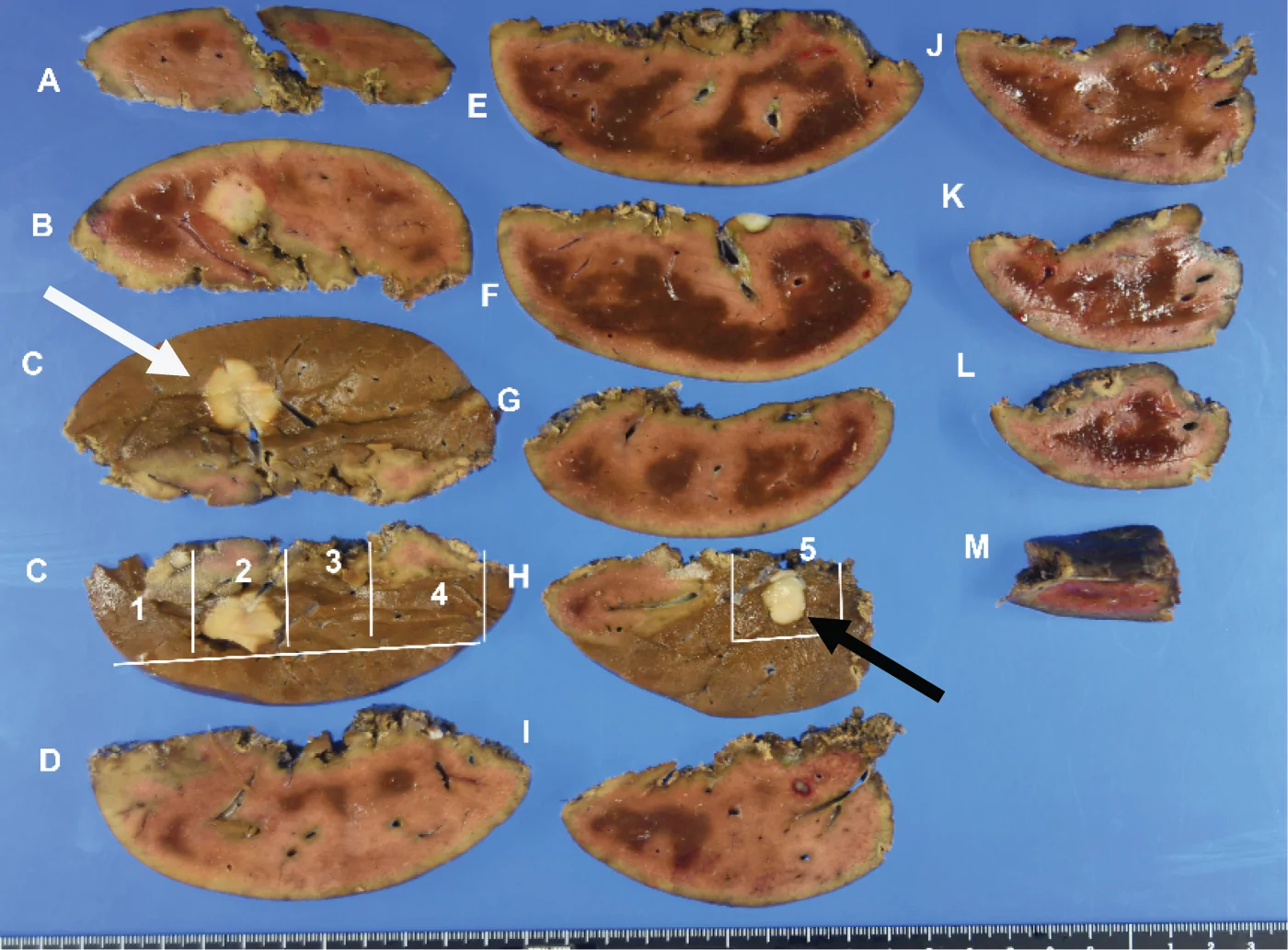
Resected specimen showed 2 white nodules measuring approximately 20 mm (white arrow) and 9 mm (black arrow). No other gross lesions were identified. (The cut surfaces were labeled A–M. Tissue blocks C1–C4 and H5 were sampled for histopathological examination. The histopathological findings of tissue block H5 are shown in **Fig. 6**.)

**Fig. 6:**
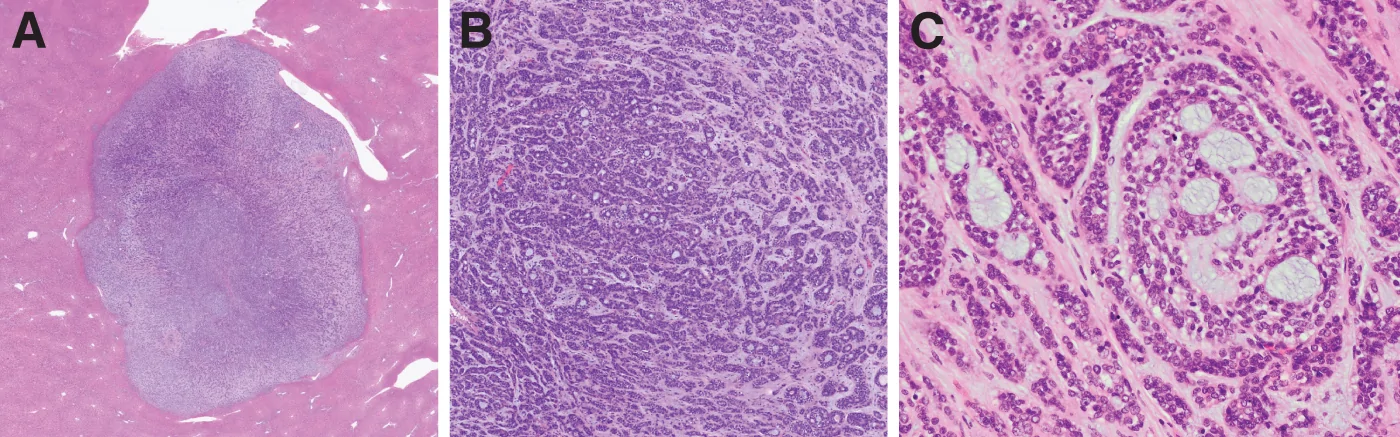
Pathological findings of the liver metastases. (**A**) Macroscopic image. (**B**) Low-magnification image (H&E, ×4). (**C**) High-magnification image (H&E, ×40). Similar to the primary tumor, the metastatic lesion was composed of tumor cells arranged in cribriform, trabecular, and tubular patterns. H&E, hematoxylin and eosin

**Fig. 7:**
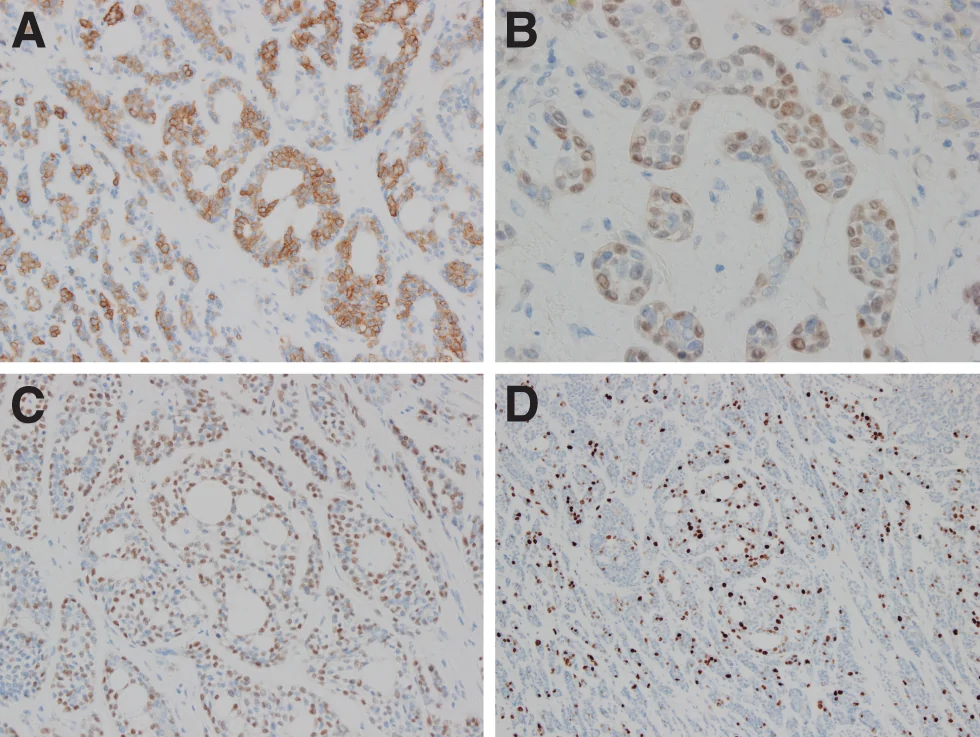
Immunohistochemical findings of the liver metastases. (**A**) c-kit (CD117), ×200. (**B**) Ki-67, ×100. (**C**) MYB, ×400. (**D**) p63, ×200. The positive results for c-kit, MYB, and p63 are consistent with liver metastases from LGACC. LGACC, lacrimal gland adenoid cystic carcinoma

Postoperative course: The patient’s postoperative course was uneventful. The patient was discharged on POD 11 without any complications. At the latest follow-up 11 months after hepatectomy, the patient remained recurrence-free.

## DISCUSSION

This case represents a rare presentation of LGACC with liver metastasis treated by surgical resection.

ACC is a rare malignancy with an incidence of approximately 3–4 cases per million individuals^2)^ and constitutes approximately 1% of all head and neck malignancies.^3)^ The standard treatment for early-stage LGACC (T1–T2) involves radical surgical resection, followed by postoperative radiotherapy, which yields favorable local control rates.^4)^ Perineural invasion is a key characteristic of ACC. Invasion of the central nervous system has also been observed in LGACC. Although the overall local recurrence rate for ACC is 26%, perineural invasion-positive cases show poor prognosis, with a local recurrence rate of 53% and a distant metastasis rate of 37%.^5)^ Another characteristic is slow progression, which results in a higher 5-year survival rate than that of cancers of other organs.

However, delayed distant metastases occur frequently, developing in approximately 40% of all cases and necessitating long-term follow-up.^1)^ Hematogenous spread is the predominant metastatic route, whereas lymphatic metastases are relatively rare. Regarding metastatic sites, Seok et al. reported that 94% of distant metastases in ACC cases were pulmonary metastases.^6)^ Furthermore, in a major review by Pal et al., among 196 patients with LGACC with distant metastasis, 80 (41%) had lung metastasis, 49 (25%) had brain metastasis, 45 (23%) had bone metastasis, and 34 (17%) had liver metastasis,^7)^ indicating that liver metastasis is relatively rare.

The optimal treatment strategy for recurrent or metastatic ACC has not yet been established. Although several systemic therapies, including vascular endothelial growth factor receptor–targeted agents and other molecular targeted therapies, have demonstrated modest antitumor activity,^8–10)^ no systemic treatment has been established as the standard of care. Therefore, treatment should be individualized according to tumor biology, metastatic burden, and the feasibility of local treatment.

The efficacy of surgical treatment has been demonstrated in selected patients with pulmonary metastases. Ishida et al. demonstrated that salvage surgery for distant metastatic ACC, which was performed predominantly for pulmonary metastases, was associated with improved disease-specific survival.^11)^ Girelli et al. reported 5- and 10-year overall survival rates of 66.8% and 40.5%, respectively, after pulmonary metastasectomy, with complete resection and a disease-free interval longer than 36 months identified as favorable prognostic factors.^12)^ These findings suggest that aggressive local treatment may benefit patients with slowly progressive metastatic ACC. However, evidence supporting local treatment remains limited. Hoff et al. recently reported that pulmonary metastasis-directed local therapy did not significantly improve overall survival compared with nonlocal treatment, indicating that patient selection remains crucial and that the benefit of local therapy should not be generalized to all patients with metastatic ACC.^13)^

Similar to pulmonary metastases, the role of hepatectomy for ACC liver metastases has not been established because of the rarity of the disease. Nevertheless, Zheng et al. suggested that surgical resection or local ablative therapy may achieve long-term survival in selected patients with resectable liver metastases.^14)^

To better characterize patients who may benefit from hepatectomy, we reviewed 15 previously reported cases of surgically resected liver metastases from ACC. The PubMed search was performed using the terms 「adenoid cystic carcinoma」 (ACC) 「liver resection」 from 2000 through 2026. Only surgically resected liver metastases with sufficient clinical information were included (**Table 1**).^15–27)^ The primary sites were the parotid gland (4 cases), submandibular gland (4 cases), trachea (4 cases), and lacrimal gland (3 cases). After excluding cases 6 and 10 due to synchronous metastases, the median interval from primary resection to recurrence was 72 months (range, 12–456 months), reflecting the indolent biological behavior of ACC. Notably, case 9 achieved a recurrence-free survival of 44 months following local treatment consisting of hepatectomy combined with radiofrequency ablation (RFA), despite the presence of 7 liver metastases. Furthermore, case 8 achieved a recurrence-free survival of 24 months after surgical resection despite having metastases involving multiple organs. These findings suggest that aggressive local treatment may provide durable disease control in carefully selected patients with ACC, even in the presence of multiple liver lesions or limited extrahepatic metastases.

**Table 1:** Surgical resection cases for recurrent liver metastases from ACC

Case	Author	Publication year	Age	Sex	Primary site	Number of metastatic lesions	Time to liver metastasis (months)	Surgical procedure	Surgical margin	Recurrence-free survival (months)	Recurrence after hepatectomy (site)
1	Scuderi^15)^	2000	30	F	Parotid gland	Solitary	132	Left hepatectomy	R0	21	Yes (Liver)
2	Bashar^16)^	2006	71	M	Lacrimal gland	Solitary	240	Extended right hepatectomy	R0	N.A.	N.A.
3	Balducci^17)^	2006	55	M	Submandibular gland	Multiple (4 sites)	18	Extended right hepatectomy	R0	18	Yes (Liver)
4	Park^18)^	2009	52	F	Trachea	Multiple (2 sites)	36	Right hepatectomy	R0	24	No
5	Zemni^19)^	2013	29	F	Parotid gland	Solitary	60	Partial resection	R0	12	No
6	Spolverato^20)^	2014	59	F	Submandibular gland	Solitary	–	Left hepatectomy	R0	5	No
7	Coupland^21)^	2014	52	F	Parotid gland	Solitary	456	Segmentectomy	R0	6	No
8	Sali^22)^	2016	42	F	Lacrimal gland	Multiple (2 sites) (+Lung)	48	Right hepatectomy (→Lung partial resection)	R0	24	No
9	Hashimoto^23)^	2018	77	M	Trachea	Multiple (7 sites)	168	Extended right posterior sectionectomy +Partial resection+RFA	–	44	Yes (Kidney, Peritoneum)
10	Hashimoto^23)^	2018	53	F	Trachea	Multiple (2 sites)	–	Partial resection	R0	12	No
11	Jing^24)^	2020	69	F	Submandibular gland	Solitary	132	Left hepatectomy	R0	24	No
12	Nakashima^25)^	2021	76	M	Submandibular gland	Solitary	60	Extended posterior sectionectomy	R0	6	No
13	Pasquale^26)^	2023	54	M	Larynx/Trachea	Solitary	12	Left hepatectomy	R0	3	No
14	Desai^27)^	2024	32	F	Parotid gland	Multiple (>8 sites)	84	Right hepatectomy	R0	6	No
15	Present case	2026	38	M	Lacrimal gland	Multiple (2 sites)	72	Right anterior sectionectomy	R0	11	No

Cases 6 and 10 were excluded from the analysis of time to liver metastasis.

ACC, adenoid cystic carcinoma; F, female; M, male; RFA, radiofrequency ablation; N.A., not available

The present patient had previously undergone pulmonary metastasectomy. Nevertheless, all metastatic lesions had been controlled by local treatment, and no uncontrolled extrahepatic disease was identified at the time of hepatectomy. Furthermore, the patient had a long clinical course, favorable performance status, limited hepatic tumor burden, and technically resectable disease. Considering the absence of an established effective systemic therapy for metastatic ACC, curative-intent hepatectomy was considered an appropriate treatment strategy in this carefully selected patient.

In conclusion, although the optimal management of metastatic ACC remains controversial, hepatectomy may represent a reasonable treatment option for carefully selected patients with indolent tumor biology, limited liver metastases, and resectable disease.

## CONCLUSIONS

Herein, we report a rare case of liver metastasis from LGACC treated with curative surgical resection. The patient achieved a favorable postoperative course with no recurrence at 11 months. Surgical resection may be considered a potential treatment option for selected patients with resectable liver metastases from ACCs.

## DECLARATIONS

### Availability of data and materials

The datasets used and/or analyzed during the current study are available from the corresponding author on reasonable request.
